# Phylogenetic grouping, epidemiological typing, analysis of virulence genes, and antimicrobial susceptibility of *Escherichia coli* isolated from healthy broilers in Japan

**DOI:** 10.1186/2046-0481-67-14

**Published:** 2014-07-10

**Authors:** Mototaka Hiki, Masaru Usui, Tetsuo Akiyama, Michiko Kawanishi, Mai Tsuyuki, Saiki Imamura, Hideto Sekiguchi, Akemi Kojima, Tetsuo Asai

**Affiliations:** 1 National Veterinary Assay Laboratory, Ministry of Agriculture, Forestry and Fisheries, 1-15-1 Tokura, Kokubunji, Tokyo 185-8511, Japan; 2 Laboratory of Food Microbiology and Food Safety, Department of Health and Environmental Sciences, School of Veterinary Medicine, Rakuno Gakuen University, Ebetsu, Hokkaido, Japan; 3 Food Safety and Consumer Affairs Bureau, Ministry of Agriculture, Forestry and Fisheries, 1-2-1 Kasumigaseki, Chiyoda-ku, Tokyo 100-8950, Japan; 4 The United Graduate School of Veterinary Sciences, Gifu University, Gifu-shi, Gifu, Japan

**Keywords:** Broiler, *Escherichia coli*, Multi-locus sequence typing, Phylogenetic grouping, Virulence-associated gene, Antimicrobial resistance

## Abstract

**Background:**

The aim of our study was to investigate the possible etiology of avian colibacillosis by examining *Escherichia coli* isolates from fecal samples of healthy broilers.

**Findings:**

Seventy-eight *E. coli* isolates from fecal samples of healthy broilers in Japan were subjected to analysis of phylogenetic background, virulence-associated gene profiling, multi-locus sequence typing (MLST), and antimicrobial resistance profiling. Phylogenetic analysis demonstrated that 35 of the 78 isolates belonged to group A, 28 to group B1, one to group B2, and 14 to group D. Virulence-associated genes *iutA*, *iss*, *cvaC*, *tsh*, *iroN*, *ompT*, and *hlyF* were found in 23 isolates (29.5%), 16 isolates (20.5%), nine isolates (11.5%), five isolates (6.4%), 19 isolates (24.4%), 23 isolates (29.5%), and 22 isolates (28.2%) respectively. Although the genetic diversity of group D isolates was revealed by MLST, the group D isolates harbored *iutA* (10 isolates, 71.4%), *iss* (6 isolates, 42.9%), *cvaC* (5 isolates, 35.7%), *tsh* (3 isolates, 21.4%), *hlyF* (9 isolates, 64.3%), *iroN* (7 isolates, 50.0%), and *ompT* (9 isolates, 64.3%).

**Conclusions:**

Our results indicated that *E. coli* isolates inhabiting the intestines of healthy broilers pose a potential risk of causing avian colibacillosis.

## Findings

*Escherichia coli* is a commensal bacterium found in the intestinal microflora of animals. *E. coli* clones that carry and acquire specific virulence attributes can cause a broad spectrum of diseases. Avian pathogenic *E. coli* (APEC), the etiological agent of avian colibacillosis, causes extraintestinal infections — primarily respiratory infections, pericarditis, and septicemia in poultry. Avian colibacillosis was thought to be an opportunistic infection predisposed by stress [1]. Although the origin of APEC is not clearly defined, we speculated that APEC may originate from the healthy host’s own or the same flocks’ fecal flora.

The phylogenetic grouping of *E. coli* for the classification of extraintestinal pathogenic strains (groups B2 and D) and commensal strains (groups A and B1) in humans [2] has been applied to the characterization of *E. coli* strains from poultry. Previous studies have shown that group A and group D were predominant in APEC in Japan [3] and the United States [4]. On the other hand, Jakobsen *et al*. reported that only group A (approximately 40%, non-typeable was counted as group A as was done in this study) was the dominant phylogenetic group among *E. coli* from healthy broilers (n = 138) [5]. Thus, the proportion of the phylogenetic groups might be different between APEC and *E. coli* from healthy broilers.

Some virulence genes are frequently found in APEC and assumed to be related to avain colibacillosis. *iutA* and *iroN* are iron transporter-encoding genes linking with growth in iron-poor environment. *cvaC*, *iss,* and *ompT* are associated with serum and/or complement resistance linking to systemic infection. *tsh* is associated with pathogenic process. *hlyF* is associated with toxin [6-8]. However, the pathogenesis and the role of virulence genes in avian colibacillosis have been obscure. Therefore, prevalence of these genes was analyzed as the virulence-associated genes in *E. coli* isolates from healthy broilers in this study, to compare with that of APEC in Japan.

Molecular analyses, such as multi-locus sequence typing (MLST) of *E. coli*[9,10], may provide additional epidemiological information on *E. coli* isolates from healthy broilers when used in combination with phylogenetic grouping or virulence profiling.

We have revealed previously that the resistance rates to ampicillin and enrofloxacin of APEC strains were higher than those of *E. coli* isolated from healthy broilers, suggesting that the antimicrobial resistance profile of APEC was different from that of *E. coli* isolated from healthy broilers. Furthermore, several studies have shown that phylogenetic groups were related to the antimicrobial resistance of *E. coli* isolates of human origin [5,11-13].

The aim of our study was to investigate possible associations of *E. coli* isolates from fecal samples of healthy broilers with APEC in Japan using phylogenetic grouping, virulence-associated gene profiling, MLST, and antimicrobial resistance profiling.

## Material and methods

### Bacteria

Ninety-six *E. coli* isolates were collected from 53 fecal samples from healthy broilers in 22 prefectures across Japan under the Japanese Veterinary Antimicrobial Resistance Monitoring System (JVARM) in 2009. Fresh fecal samples were collected from two or three healthy broilers at the different farms in each prefecture as previously described [14]. *E. coli* was isolated from the fecal samples using desoxycholate–hydrogen sulfate–lactose agar (Eiken Co., Ltd., Japan). Candidate colonies were identified biochemically using a commercially available kit (API20E, bioMe’rieux, Marcy-l’Etoile, France). The isolates were then stored in 10% skimmed milk (Wako Pure Chemical Industries, Ltd., Japan) at -80°C until use.

### Genotyping

The phylogenetic group (A, B1, B2, and D) of each isolate was determined by multiplex PCR as described by Clermont *et al.*[2]. Detection of the seven virulence-associated, *iutA*[15], *iss*[8], *cvaC*[8], *tsh*[15], *hlyF*[6], *iroN*[6], and *ompT*[6], was performed by PCR. Sequence type (ST) was determined using the Achtman typing scheme [16].

### Antimicrobial susceptibility test

Minimum inhibitory concentrations (MICs) were determined by the agar dilution method according to the guidelines of the Clinical and Laboratory Standards Institute (CLSI) [17]. The following antimicrobial agents were tested: ampicillin, cefazolin, ceftiofur, dihydrostreptomycin, gentamicin, kanamycin, oxytetracycline, chloramphenicol, colistin, nalidixic acid, enrofloxacin, and trimethoprim. The resistance breakpoints for cefazolin, ceftiofur, dihydrostreptomycin, kanamycin, colistin, oxytetracycline, nalidixic acid, enrofloxacin, and trimethoprim were microbiologically defined as described previously [18], and those for the other antimicrobials were defined according to CLSI guidelines [17].

### Statistical analysis

Differences in the prevalence of virulence-associated genes and the antimicrobial resistance rate between phylogenetic groups were analyzed using Fisher’s exact test, disregarding group B2 because only a single isolate was classified into this group. A value of P < 0.05 (two-sided) was considered statistically significant.

## Results and discussion

When two isolates from a fecal sample exhibited the same ST and antimicrobial resistance type, they were considered to be a single isolate. Accordingly, 78 *E. coli* isolates were used for further analyses.

Of the 78 isolates, 35 (44.9%), 28 (35.9%), one (1.3%), and 14 (17.9%) isolates belonged to groups A, B1, B2, and D, respectively (Table 1). Thus, groups A and B1 accounted for approximately 80% (63/78) of the strains isolated from healthy broilers. Our previous report showed that only 53.9% (48/89) of APEC strains from colibacillosis-affected broilers belonged to those groups [3] and this proportion was significantly low compared with this study (p = 0.0003). In addition, the proportion (17.9%) of group D isolates from the healthy broilers examined in this study was lower than that (44.9%) previously isolated from APEC-infected broilers [3]. The proportion of the phylogenetic groups in *E. coli* isolated from healthy broilers in this study is different from that in the APEC strains in Japan.

**Table 1:** **Virulence profiles and antimicrobial resistance of *E. coli *** **from healthy broilers**

	**No. (%) of positive strains**				
**Virulence-associated gene/antimicrobial**	**A**	**B1**	**B2**	**D**	**Total**
	**(n = 35)**	**(n = 28)**	**(n = 1)**	**(n = 14)**	**(n = 78)**
*iutA*	6 (17.1)^a^	7 (25.0)^a^		10 (71.4)^b^	23 (29.5)
*iss*	2 (5.7)^a^	8 (28.6)		6 (42.9)^b^	16 (20.5)
*cvaC*	2 (5.7)^a^	2 (7.1)^a^		5 (35.7)^b^	9 (11.5)
*tsh*	1 (2.9)	1 (3.6)		3 (21.4)	5 (6.4)
*iroN*	4 (11.4)^a^	8 (28.6)		7 (50.0)^b^	19 (24.4)
*hlyF*	4 (11.4)^a^	9 (32.1)		9 (64.3)^b^	22 (28.2)
*ompT*	5 (14.3)^a^	9 (32.1)		9 (64.3)^b^	23 (29.5)
Ampicillin	16 (45.7)	10 (35.7)	1	7 (50.0)	34 (43.6)
Cefazolin	6 (17.1)	4 (14.3)		4 (28.6)	14 (17.9)
Ceftiofur	4 (11.4)	4 (14.3)		4 (28.6)	12 (15.4)
Dihydrostreptomycin	11 (31.4)	10 (35.7)		7 (50.0)	28 (35.9)
Gentamicin	2 (5.7)	1 (3.6)			3 (3.8)
Kanamycin	7 (20.0)	2 (7.1)		3 (21.4)	12 (15.4)
Oxytetracycline	19 (54.3)	13 (46.4)	1	9 (64.3)	42 (53.8)
Chloramphenicol	5 (14.3)	4 (14.8)		1 (7.1)	10 (12.8)
Colistin	1 (2.9)				1 (1.3)
Nalidixic acid	11 (31.4)	10 (35.7)		9 (64.3)	30 (38.5)
Enrofloxacin	5 (14.3)	4 (14.3)		1 (7.1)	10 (12.8)
Trimethoprim	9 (25.7)	12 (42.9)		3 (21.4)	24 (30.8)

A significant difference (P < 0.05) in prevalence was observed between ^a^ and ^b^.

*iutA*, *iss*, *cvaC*, *tsh*, *iroN*, *ompT*, and *hlyF* were found in 23 isolates (29.5%), 16 isolates (20.5%), nine isolates (11.5%), five isolates (6.4%), 19 isolates (24.4%), 23 isolates (29.5%), and 22 isolates (28.2%) respectively (Table 1). *iutA* and *cvaC* were more frequently associated with group D isolates than groups A (P = 0.001 and 0.015 ) and B1 (P = 0.007 and 0.031) isolates, whereas *iss, hlyF*, *iroN* and *ompT* were more frequently associated with group D isolates than group A (P = 0.004, 0.0004, 0.007, and 0.001) isolates. Group D, which is the dominant phylogenetic group in APEC strains in Japan [3], may show pathogenicity to poultry compared with the commensal groups, such as group A and B1.

All the 78 isolates were classified into 46 STs (Table 2). Among the 46 STs identified in this study, six (ST10, 69, 93, 101, 117, and 155) were previously reported in APEC isolates [9,19]. In this study, among the 28 isolates belonging to the six STs, *iutA*, *iss*, *cvaC*, *tsh*, *hlyF, iroN*, and *ompT* were detected in 10 isolates (35.7%), eight isolates (28.6%), four isolates (14.3%), one isolate (3.6%), 10 isolates (35.7%), 9 isolates (32.1%), and 10 isolates (35.7%) respectively. In particular, ST117 was reported in APEC isolated in Japan [9]. All ST117 strains belonged to group D and possessed virulence-associated genes (Table 2). These results reinforced the hypothesis that the intestinal tract of healthy broilers might be an important natural reservoir for APEC.

**Table 2:** **Sequence types of *E. coli *** **from healthy broilers by virulence-associated gene profiling**

**Phylogroup**	**Virulence-associated gene**	**N**	**ST (Total no.)**
A	*iutA-iss-cvaC-tsh-iroN-ompT-hlyF*	1	1421 (1)
	*iutA-cvaC-iroN-ompT-hlyF*	1	1564 (1)
	*iss-iroN-ompT-hlyF*	1	48 (1)
	*iroN-ompT-hlyF*	1	1286 (1)
	*iutA*	4	10 (1)^a^, 48 (1), 93 (1)^a^, 2469 (1)
	*ompT*	1	ND (1)*
	None	26	10 (11)^a^, 752 (1), 1286 (1), 1630 (1), 2223 (2), 2461 (1), 2462 (1), 2463 (2), 2465 (1), 2466 (2), 2470 (1), 2471 (1), 2378 (1)
B1	*iutA-iss-cvaC-iroN-ompT-hlyF*	1	58 (1)
	*iutA-iss-tsh-iroN-ompT-hlyF*	1	1079 (1)
	*iss-cvaC-iroN-ompT-hlyF*	1	154 (1)
	*iutA-iss-iroN-ompT-hlyF*	2	155 (1)^a^, 156 (1)
	*iss-iroN-ompT-hlyF*	3	155 (3)^a^
	*iutA-ompT-hlyF*	1	101 (1) ^a^
	*iutA*	2	155 (1)^a^, 2472 (1)
	None	17	155 (4)^a^, 295 (1), 453 (2), 533 (1), 641(1), 1079 (1), 1724 (1), 1730 (1), 1771 (1), 2464 (1), 2473 (1), 2475 (1), 2476 (1)
B2		1	2474 (1)
D	*iutA-iss-cvaC-tsh-iroN-ompT-hlyF*	1	69 (1)^a^
	*iutA-iss-cvaC-iroN-ompT-hlyF*	2	117 (2)^a^
	*iss-cvaC-tsh-iroN-ompT-hlyF*	1	196 (1)
	*iutA-cvaC-iroN-ompT-hlyF*	1	117 (1)^a^
	*iutA-tsh-iroN-ompT-hlyF*	1	57 (1)
	*iutA-iss-iroN-ompT-hlyF*	1	117 (1)^a^
	*iutA-iss-ompT-hlyF*	1	2309 (1)
	*iutA-ompT-hlyF*	1	2477 (1)
	*iutA*	2	350 (1), 420 (1)
	None	3	297 (2), 457 (1)

*ND: not determined because the gene for MLST could not be amplified by PCR.

a: common STs of APEC isolates.

A difference in the resistance rates between APEC strains of the previous study [10] and *E. coli* isolated from healthy broilers of this study was observed. The resistance rates against ampicillin (P = 0.00002), kanamycin (P = 0.006), and oxytetracycline (P = 0.005) of *E. coli* isolated from healthy broilers were significantly lower than those of APEC [10]. Our previous study showed that the resistance rates against ampicillin and enrofloxacin were higher in APEC strains than in isolates from healthy broilers [10]. Although the higher resistance rate to enrofloxacin was not observed in this study, the 1116.7-kg enrofloxacin was used for the treatment of broilers in 2009 (National Veterinary Assay Laboratory, 2009). In Japan, oxytetracycline has been widely used for the treatment of bacterial infection in poultry, ampicillin and kanamycin as well as enrofloxacin have been approved for the treatment of avian colibacillosis.

## Conclusion

We demonstrated that the proportion of the phylogenetic groups in *E. coli* isolated from healthy broilers is different from that in APEC strains in Japan. On the other hand, even though group D strains accounted for a minor portion of *E. coli* isolates from healthy broilers, group D strains frequently possessed virulence-associated genes. Thus, *E. coli* isolates from the intestines of healthy broilers pose a potential risk of causing avian colibacillosis.

## Competing interests

The authors declare that they have no competing interests.

## Authors’ contributions

MH conceived the study and the study design, interpreted the data, and drafted the manuscript. MU helped draft the manuscript. TA helped carry out the determination of phylogenetic groups and virulence-associated genes. MK, MT, SI, HS, AK, and TA helped draft the manuscript. All authors have read and approved the final manuscript.
